# Challenges for health care providers, parents and patients who face a child hood cancer diagnosis in Zambia

**DOI:** 10.1186/s12913-018-3127-5

**Published:** 2018-05-02

**Authors:** Mulima Walubita, Bornwell Sikateyo, Joseph M. Zulu

**Affiliations:** School of Public Health, Department of Health Promotion and Education, University of Zambia, P.O. Box 50110, Lusaka, Zambia

**Keywords:** Childhood cancer diagnosis, Challenges, Zambia

## Abstract

**Background:**

Zambia is experiencing high prevalence of childhood cancer. However, very few children access and complete treatment for cancer. This study aimed to document the challenges for health care providers, parents and patients who face a child hood cancer diagnosis in Zambia, and their coping strategies.

**Methods:**

This was an exploratory health facility-based qualitative study that was conducted at a Paediatric oncology ward at referral hospital in Zambia. In-depth individual interviews conducted with fifteen (15) caregivers and seven (7) key informants were analysed using thematic analysis.

**Results:**

Several challenges related to managing the childhood cancer diagnosis were recorded. Individual and family challenges were inadequate knowledge on childhood cancer, lack of finances to meet treatment and transport costs as well as long period of hospitalisation that affected women’s ability to perform multiple responsibilities. Whereas challenges at community level were inadequate support to address emotional and physical distress and social stigmatisation experienced by caregivers. Health systems issues included inadequate specialised health workers, poor communication among health workers, limited space and beds as well as insufficient supplies such as blood. Cultural related factors were the belief that cancer is a product of witchcraft as well as religious beliefs regarding the role of faith healing in childhood cancer treatment. Coping strategies used by parents/ caregivers included praying to God, material support from organisations and church as well as delaying having another child.

**Conclusion:**

Addressing the challenges for health care providers, parents and patients who face a childhood cancer diagnosis may require adopting a systems or an ecological approach that allows developing strategies that simultaneously address challenges related to the individual, family, community, health system and cultural aspects.

**Electronic supplementary material:**

The online version of this article (10.1186/s12913-018-3127-5) contains supplementary material, which is available to authorized users.

## Background

Cancer is the leading cause of death in developed countries and the second leading cause of death in developing countries [1–3]. About 12.7 million new cases of cancer occur each year worldwide and 56 % of the cases occur in low and middle-income countries (LMICs) [1–3]. A significant number of these cancer cases involve children [3]. Globally, about 160,000 new cases of cancer are diagnosed annually in children younger than 15 years of age [1–3]. Sub-Saharan Africa has faced a huge challenge with regard to diagnosis and treatment of cancer in children such as difficulties in giving the right diagnosis and at the right time and reduced access to appropriate therapeutic facilities and drugs [4–6]. In Zambia, about 1000 cases of pediatric cancer occur per year and less than 10% of these children complete treatment [2].

Despite this increasing burden, cancer control programs and the provision of early diagnosis and treatment services are limited in most African countries [3]. Competing health demands and limited knowledge concerning the magnitude of the current and future cancer burden and its economic impact has contributed to inadequate investment in cancer control programs and the provision of services in most African countries [3, 7, 8].

There are several socio-economic and psychosocial effects of cancer on children and their families [3, 9]. To come up with sustainable support for the children with childhood cancer as well as their parents, the main needs that they face need to be considered [3, 9, 10]. Some of these needs and challenges include inadequate access to relevant information, stress, lack finances to meet the health needs and poor nutrition to support the treatment [10].

In Zambia, the University Teaching Hospital (UTH) is the only major government-funded institution offering psychosocial support and treatment services to children with cancer [2, 11]. Utilization of these services is very poor [2]. Meanwhile, there is limited documentation on the challenges faced by health care providers, parents and patients who face a child hood cancer diagnosis. Current studies on childhood cancer in Zambia have mainly focused on the effects of the HIV epidemic on the epidemiology of cancers in children [12]; and an investigation of treatment outcomes and risk factors for treatment abandonment of children diagnosed with cancer [2]. This study aimed at addressing this knowledge gap by documenting the challenges and coping strategies for health care providers, parents and patients who face a child hood cancer diagnosis in Zambia at UTH.

## Methods

### Study design

This was an exploratory health facility-based qualitative study. It involved parents or caregivers of children with cancer undergoing treatment at the Paediatric oncology ward at UTH in Lusaka district. The study design helped in bringing forth in-depth insights on perspectives of parents/ caregivers on psychosocial support needs at the Paediatric oncology ward at UTH. The research question was: what are the challenges for health care providers, parents and patients who face a child hood cancer diagnosis in Zambia? To adequately capture the challenges for all the categories in the research question, the interview guide explored the challenges at individual, family, community, systems and cultural levels.

### Study setting

The study was conducted at the Paediatric oncology ward at UTH in Lusaka district, the capital city of Zambia. The site was selected because it is the only health facility that offers paediatric cancer treatment in Zambia. The UTH is a 2000-bed tertiary care institution and serves as the country’s principal referral hospital. In the UTH’s Department of Paediatrics and Child Health, the Haematology-Oncology Unit has a 32 bed-capacity and offers chemotherapy. Radiation therapy became available in 2006 at UTH. However, there remain inadequate human resources to provide adequate services with the nurse to patient ratio of 1:15. There is only one subspecialty trained paediatric haematologist-oncologist in Zambia. As of 2014, immunohistochemistry, cytology, and molecular diagnostics were not available and imaging modalities were limited with one Magnetic Resonance Imaging (MRI) and two Computerized Tomography (CT) imagers that serve the entire country, thus negatively impacting initial diagnosis or follow-up strategies [11].

Zambia is a lower middle-income country. In 2013, the country had a Gross Domestic Product (GDP) of about 17 3751. 82 million United States Dollar (USD) and a GDP per capita of 1115 (USD). The country has a population of about 14.5 million and half are aged below 15 years. About 60% of the population is below the international poverty line of USD 1.25 per day. The Zambian health system faces multiple challenges. HIV prevalence in the adult Zambian population stands at 12.7%. The average life expectancy in Zambia is 51.2 years, while the neonatal mortality rate is 35 per 1000 live births. Infant and under-five mortality are also high, at 86 and 141 per 1000 live births, respectively [13].

### Sampling and sample size

The sample size was 22 respondents: 7 key informants and 15 parents/caregivers. Parents/caregivers were purposively recruited from patient register at the ward over a period of month. We approached all those who were in the Paediatric oncology ward to seek informed consent. The recruitment process started with explaining the purpose and scope of study to the study participants. All the parents/caregivers that we approached agreed to participate in the study. The final sample was arrived at using maximum-variation sampling criteria, which was about choosing parents/caregivers with different characteristics based on demographic characteristics such as age, marital status and district of residence until data saturation was reached [13].

The inclusion criteria were a parent/caregiver whose child had been diagnosed and admitted in the ward, as well as workers who provided treatment to children and psychosocial support to parents/guardians. We also included officials from Non-Governmental Organizations providing psychosocial support services to the ward. We excluded all those who met the inclusion criteria but did not provide consent.

### Data collection

Data were collected using different methods namely in-depth interviews (Additional file 1), key informant interviews (Additional file 2) and review of documents. Interview guides were developed to guide data collection. The in-depth interview guide was administered through face-to-face in-depth individual interviews with parents/caregivers. Interviews were recorded using a digital audio recorder. Handwritten interview notes were also taken in an interview notebook. Data collection and preliminary data analysis was a cyclical process: data collected were informing ensuing interviews and data collection was concluded when no new insight emerged, a stage called data saturation.

The key informant interviews (KII) were conducted with five health professionals from UTH: a social worker, pharmacist, registered nurse (trained counsellor), an enrolled nurse, and an oncology doctor as well as two staff from Kayula Childhood Cancer Foundation (KCCF) and Zambia Childhood Cancer Foundation (ZCCF) who provide psychosocial support services at the Paediatric oncology ward at UTH. The KII provided insights into the challenges experienced in providing services. The officials had varying years of experience ranging from 1 year 6 months to 13 years.

### Secondary data review

Secondary data review helped in providing an-in depth understanding on psychosocial support services available at the Paediatric oncology ward at UTH. We also reviewed published literature on childhood cancer in Zambia.

### Data analysis

All interviews were tape recorded, and transcribed verbatim. The transcripts were stored on a password-protected computer with only access to researcher. Data were analysed using thematic analysis. The transcripts were coded and read several times in order to make sense of the conversation in the transcripts. The codes were compared for similarities and differences by conducting within-and across-case analysis. Similar codes were then grouped together to form categories and then themes were developed by interpreting the categories for their underlying meaning [14, 15].

### Ethical considerations

Ethical clearance was sought from the Excellency in Research Ethics and Science (ERES) commitee, clearance number 2014-May-031. Permission was obtained from UTH authorities. The study was explained to the participants and written informed consent was sought from the study participants before they took part in the study. All the 26 participants whom we approached agreed to participate in the study. We could not interview four participants who initially agreed to be part of the study as three of them were discharged from the ward while the child of one of the potential respondents died during the study period. The interviews were not conducted in the presence of the child but in private spaces within the health facility to ensure both confidentiality and openness. Identification of a participant was only done through numerical codes. To address the possible effects of emotional breakdown (crying) during the interview, caution was taken by having counsellors from the UTH counselling centre, the counsellor at the Paediatric oncology ward and the social worker (who is also a counsellor) on standby for any support.

## Results

### Socio-demographic characteristics

A total of 15 parents/ caregivers to children with cancer were interviewed. Parents/ caregivers’ age ranged from 20 years to above 58 years (Table 1). Fourteen parents/caregivers were female and only one (1) was male. In terms of marital status, eight (8) were married, four (4) widowed, one (1) was single, one (1) divorced, and one (1) widower.

**Table 1:** Type and sex of parent / guardian

Type of Parent/Guardian		Sex	
		Female	Male
Biological	12	11	1
Grandparent	2	2	0
Great grand parent	1	1	0
Totals	15	14	1

The age for the childhood cancer patients ranged from 2 to 14 years, and they comprised both male and female (Table 2). Fourteen (14) out of fifteen (15) parents were already aware of diagnosis. Only one (1) parent was still awaiting diagnosis. Of the fifteen (15) children, four (4) children were diagnosed with cancer of the kidney, three (3) with leukaemia, another three (3) with retinol blastoma, two (2) with lymphoma, one (1) with cancer of the liver, another one (1) with cancer of the lungs. Fourteen (14) out of the fifteen (15) parents interviewed said their children were receiving treatment. One (1) child was not receiving treatment due to low levels of blood and platelets the child was experiencing.

**Table 2:** Age and sex of childhood cancer patient

Age	Female	Male	Totals
0–1 year	0	0	0
2–5 years	6	3	9
6–10 years	3	2	5
11–14 years	0	1	1
Totals	9	6	15

### Challenges related to child hood cancer diagnosis in Zambia

In this section we have categorized the challenges for health care providers, parents and patients who face a child hood cancer diagnosis under the following major themes: individual and family factors, community factors, health system factors, cultural factors and coping strategies.

### Inadequate knowledge on childhood cancer among parents/care givers

Inadequate knowledge on childhood cancer was one of the individual level challenges. Most of the parents/ caregivers exhibited lack of knowledge on the symptoms of childhood cancers before diagnosis of the cancer. This lack of knowledge contributed to caregivers presenting the children late at clinics/hospitals. Most of the parents/guardians reported that they only heard about childhood cancer after diagnosis at the Paediatric oncology ward at UTH.

“*I never heard about cancer in children before they told me that my son had cancer”* (30–35 years).

### Household poverty

Lack of finances at family level made it difficult for parents to meet transport costs to the hospital and food costs. As a result of stress of nursing patients and staying away from home during the process of treatment, many parents/ guardians failed to continue with the various income generating activities that they were involved in. Their children’s condition had negatively affected their productivity and they felt depressed. They also worried a lot about the welfare of children back home and their spouses.

*“I have had financial challenges, all the tests that are done I have to pay, and I have a home to take care of, I am a widow”* (35–40 years)*.*

### Stigmatisation and discrimination at community level

At community level, some parents complained of being neglected or isolated once their child was diagnosed with cancer. The feeling of lack of support was common among widows, widowers, divorcees, and single parents. They felt that having a spouse to help nurse the child with cancer would lighten the burden.

*“I have a problem because my relatives don’t help me”* (35–40 years).

In some cases, parents were not happy with friends and community members talking about the child’s illness in their absence. Some parents were accused of infecting the child with the cancer by some of the community members.

*“They think that I am responsible for the child’s illness”* (20–25 years).

### Health system gaps and child hood cancer diagnosis

Inadequate availability of specialised health workers was one of the health systems challenges. The number of patients and parents/caregivers to counsel is very high as compared to the number of counsellors and doctors at the ward. This shortage resulted into inadequate provision of counselling services.

“*The huge number of parents and patients is overwhelming compared to staff. Time is limited and we do not have sufficient time to attend to every patient and parent”* (Key Informant 2).

Additional human resources for health problems included poor attitude among some health workers. Parents reported that some nurses did not pay adequate attention to the patients. Some health workers faced difficulties in providing counselling to parents/caregivers of children with cancer as some parents could not speak English but only local languages that are different from those spoken by the health workers.

Inadequate availability of supplies was another health system challenge. Parents for example complained of the shortage of blood at UTH blood bank. They reported that this shortage was a great source of concern as it is a risk on the children’s lives. Other parents/ caregivers were concerned with inadequate bed spaces at the hospital. As a result, some parents/caregivers together with the sick child would sleep on the floor.

“*. Since I came back on Wednesday I have been sleeping on the floor with the child. Bed spaces are really few*” (20–21 years).

### Cultural beliefs regarding the cause and treatment of cancer

The cultural belief among some parents that childhood cancer is a result of witchcraft affected seeking of medication. Due to these cultural beliefs, some parents/guardians were not willing to place their children on chemotherapy as they opted for traditional medicine such as indigenous herbalism through the use of leaves, seeds, twigs and tree barks.

*“ Some of them don’t want to be counselled, they want to leave the wards as they believe that the solution lies in the traditional healers*” (Key Informant 1).

Religion is another cultural issue which affected access to cancer treatment. It was reported that some pastors teach doctrines that discourage parents/caregivers from seeking medical attention in preference for faith healing. The pastors convince parents/caregivers to believe that their children are cancer free after a series of healing prayers.

*“The other challenge is with the churches that are not teaching the right doctrine where pastors tell the parent that their child has been healed after prayers even when the child still has cancer”* (Key Informant 1).

### Coping strategies

Copying strategies at individual level included talking to friends about other stories that have nothing to do with childhood cancer. Taking to other parents helped them not to think negatively about the sick child’s condition. Some parents/caregivers put child bearing on hold. They avoided having more children so that they can concentrate on nursing the sick child.

“*I went for an injection (family planning) for 5 yrs. so that I don’t get pregnant and have time to look after the child who is not well*” (20–25 years).

At community level, the main coping strategy was support from fellow caregivers within the hospital, family, friends, church members. The support was mainly in form of material support (food, clothes) and spiritual support.

“*When am here I cook vegetables and he doesn’t want vegetable but wants to eat meat and I don’t have it, so I ask from my neighbours and those who have will give me*” (20–25 years).

At systems level, the Social Welfare Department provided material support to parents and caregivers when they were in the hospital. Though not adequate, the parents and caregivers appreciated this support.

Finally, prayer was a major cultural coping strategy that parents’/caregivers depended on. During hospitalisation, parents/caregivers congregated at nearby churches where they accessed spiritual support.

“*I leave everything in God’s hands. I just put God before me so that he cures my child*” (30–35 years).

## Discussion

This study explored challenges for health care providers, parents and patients who face a child hood cancer diagnosis in Zambia. Challenges documented in this study included inadequate specialised staff and poor attitude of health workers, difficulties in meeting treatment costs, low knowledge levels on childhood cancer among parents/guardians as well as challenges in managing long periods of hospitalisation away from their home. Cultural issues such as witchcraft and religious beliefs also affected seeking of cancer treatment services. These findings are similar to other studies which have also shown that low knowledge, logistical challenges faced by families, and significant distance from the hospital contributed to low utilisation of childhood cancer preventive and treatment services [2, 3, 16–23].

Based on the findings of the study, perhaps it may be important to adopt an ecological or systems approach to understanding and addressing the challenges for health care providers, parents and patients who face a child hood cancer diagnosis in Zambia. Adopting such an approach is vital as the challenges related to child cancer diagnosis are intertwined and may require strategies that simultaneously responded to the challenges at multiple layers (individual, community, systems and cultural levels) at the same time [2, 3, 9, 17].

At the individual level, strategies for enhancing access to diagnosis, prevention and treatment of cancer services may focus on empowering people with knowledge on childhood cancer, providing psychosocial support, and developing safe spaces. Other studies have also shown that parents wanted to learn about the illness by talking to health care providers, to other parents, and through using the internet [24, 25]. At the relationship or family level, strategies may aim to enhance social support networks. At the community level it may be important to invest in health education aimed at transforming gender regimes that negatively affect the welfare of female caregivers as well as other social norms and beliefs that trigger stigmatisation of cancer related matters. In a study conducted by Van Dongen-Melman [26], medical and psychosocial information was considered crucial to reducing uncertainty and aiding patient and parents in reaching an understanding of the long-term medical and psychosocial consequences of childhood cancer [26]. At health systems and societal levels, it is vital to strengthen health education policy on childhood cancer diagnosis and overall health systems factors such as supplies, financing, service delivery as well as availability and attitude of health workers. Grootenhuis and Las found that personal contact with health workers enabled families/parents to develop relationships with staff that in turn enhanced the level of satisfaction in terms of treatment offered [24].

### Limitations and strengths

Limitations of the study included some parents/caregivers not completing interviews due to the critical condition of their children. It was not possible to follow some parents due to the demise of the child. In addition, we did not collect information on distribution of diagnoses in the ward during the study period. One major strength of the study was the use of multiple methods such as key informant interviews, in-depth interviews and review of records that enabled triangulation of the information.

## Conclusions

This study provided context specific information on the challenges for health care providers, parents and patients who face a childhood cancer diagnosis in Zambia. Challenges faced related to health systems, individual, family, community and cultural factors. Health systems challenges included inadequate human and material resources that affect delivery of quality of services. Individual level factors were insufficient knowledge and lack of finances to meet costs for accessing the services. Factors at family and community levels included multiple tasks performed by women as well as social stigmatization. Whereas dependence on traditional and faith health healing were the major cultural issues. Addressing these challenges may require developing strategies that simultaneously address barriers at the health system, individual, family or relationship, community and cultural levels. Such strategies may include strengthening health education and investing in human, material and financial resources for delivering child hood cancer services.

## Additional files

Additional file 1: In-depth interview guide for parents/caregivers (DOC 26 kb)

Additional file 2: Key informant interview guide (DOC 27 kb)

## Abbreviations

AIDS: Acquired immune deficiency syndrome
GDP: Gross domestic product
HIV: Human immune virus
KCCF: Kayula childhood cancer foundation
KII: Key informant interview
MRI: Magnetic resonance imaging
NGO: Non-Governmental Organization
TC: Computerized tomography
USD: United States Dollar
UTH: University Teaching Hospital
ZACCAF: Zambia Childhood Cancer Foundation
