# Supplementary material for: Challenges for health care providers, parents and patients who face a child hood cancer diagnosis in Zambia
Source: BMC Health Serv Res. 2018 May 2;18:314. doi: 10.1186/s12913-018-3127-5 (PMC5932785; doi:10.1186/s12913-018-3127-5)
Supplement: Supplementary file 1 — In-depth interview guide for parents/caregivers (DOC 26 kb) [file 12913_2018_3127_MOESM1_ESM.doc]

## Additional file 1*: In-depth interview guide for parents/ caregivers*

- What type of cancer does your child suffer from?
- When was the child diagnosed with cancer?
- Is the child receiving treatment? (If yes, since when?)
- When did you first hear about childhood cancer?
- Where did you hear about childhood cancer?
- Did you know/hear about it before your child was diagnosed?
- How do you manage taking care of the child?
- What challenges if any have you experienced since your child was diagnosed with cancer
- How have you been able to cope with your child’s illness?
- What is your comment about support from relatives, friends, the church etc.?
- What type of psychosocial support services do you receive here at the Paediatric oncology ward?
- Are the services easily accessible (Probe if they get them any time they need the services)?
- What do you think about the quality/adequacy of psychosocial support services that you receive here at the Paediatric oncology ward?
