# Supplementary material for: Challenges for health care providers, parents and patients who face a child hood cancer diagnosis in Zambia
Source: BMC Health Serv Res. 2018 May 2;18:314. doi: 10.1186/s12913-018-3127-5 (PMC5932785; doi:10.1186/s12913-018-3127-5)
Supplement: Supplementary file 2 — Key informant interview guide (DOC 27 kb) [file 12913_2018_3127_MOESM2_ESM.doc]

## Additional file 2 *: key informant interview guide*

- What does your work involve in relation to childhood cancer?
- According to your assessment, what are the levels of knowledge about cancer among parents before diagnosis, and after diagnosis?
- Do you provide any psychosocial services to caregivers with children with cancer?
- Do you have any deliberate awareness messages on childhood cancer for parents after diagnosis?
- What are the main psychosocial support needs of caregivers with children with cancer at the Paediatric oncology ward?
- What psychosocial support services are provided to caregivers with children with cancer at the Paediatric oncology ward?
- Are the services adequate in relation to the needs of parents with children with cancer?
- Do the services meet your professional standards (if any) that you are guided by?
- What challenges, if any, do you face in providing psychosocial services to caregivers with children with cancer at the Paediatric oncology ward?
- What should be done to enhance the psychosocial services?
